# Succinate modulates oral dysbiosis and inflammation through a succinate receptor 1 dependent mechanism in aged mice

**DOI:** 10.1038/s41368-025-00376-6

**Published:** 2025-06-10

**Authors:** Fangxi Xu, Yuqi Guo, Scott C. Thomas, Anish Saxena, Samantha Hwang, Mridula Vardhan, Xin Li

**Affiliations:** 1 Department of Molecular Pathobiology, New York University College of Dentistry, New York, NY USA; 2 Plastic Surgery, Maxillofacial and Oral Health Department, University of Virginia School of Medicine, Charlottesville, VA USA; 3 The UVA Comprehensive Cancer Center, School of Medicine, Charlottesville, VA USA; 4 Department of Neuroscience, University of Virginia School of Medicine, Charlottesville, VA USA

**Keywords:** Ageing, Oral diseases

## Abstract

Aging involves the accumulation of various forms of molecular and cellular damage over time. Key features of aging, such as mitochondrial dysfunction, dysbiosis, and oxidative stress, are closely linked and largely driven by inflammation. This study examines the role of succinate, a key metabolite produced and utilized by cells of both host and microbes, and its receptor, succinate receptor 1 (SUCNR1), in age-related oral dysbiosis and inflammation. We examined young and aged wild-type (WT) and SUCNR1 knockout (KO) mice for this analysis. Our findings revealed significant aging-associated alveolar bone loss and succinate elevation in aged WT mice, along with notable changes in the oral microbiome. Conversely, aged KO mice showed reduced bone loss, lower succinate levels, less inflammation, and better-maintained microbial function. These results suggest that SUCNR1 is crucial in influencing aging-related succinate elevation, oral dysbiosis, and inflammation. Analysis of gene families and pathways in the oral microbiome demonstrated distinct aging-related changes between WT and KO mice, with the functional potential being preserved in the KO-aged group. This study underscores the importance of succinate elevation and signaling through SUCNR1 in regulating inflammation, alveolar bone loss, and shifts in the oral microbiome, offering potential targets for therapeutic interventions in age-related oral health issues.

## Introduction

Aging is a natural and complex biological process characterized by the progressive deterioration of physiological functions and cellular structures over time^1^ The seven pillars of aging are inflammation, stem cell regeneration, macromolecular damage, stress, proteostasis, metabolism, and epigenetics.^2^ Intriguingly, the well-known aging pillars converge on inflammation since the impairment of any single pillar powers inflammation, which subsequently affects all the other pillars.^3–5^ Similarly, López-Otín et al. proposed that inflammation is an interconnection with the twelve integrative hallmarks of aging and recognized the gut microbiome as a new hallmark of aging.^6,7^ Studies have revealed the uniqueness of an individual’s microbiome and identified microbial metabolites involved in immune regulation, inflammation, and aging.^6^

Some of the metabolites normally present in both mammalian and bacterial cells are likely to play a pivotal role in the functionally related interconnections of the hallmarks and pillars of aging. Succinate, a tricarboxylic acid cycle (TCA) metabolite which normally functions as an intermediate substrate of succinate dehydrogenase (SDH) has emerged as a signaling molecule with multifaceted biological functions.^8^ SDH catalyzes the conversion of succinate to fumarate in the TCA cycle,^9,10^, and SDH deficiency leads to succinate accumulation.^11^ Moreover, accumulation of succinate in the extracellular environment in response to inflammation, metabolic stress, or dysbiosis leads to its binding to and activation of its cognate receptor, succinate receptor 1 (SUCNR1). SDH, also known as succinate-coenzyme Q reductase or respiratory complex II, is an enzyme complex found in many bacterial cells and in the inner mitochondrial membrane of eukaryotes. It is a key enzyme involved in normal mitochondrial function and the only enzyme that participates in both the TCA cycle and the electron transport chain. Indeed, the connection between SDH and aging has been previously noted. Mutations in SDH genes may directly contribute to aging.^12–14^ In contrast, strategies that lead to an increase in SDH activity, such as caloric restriction, have been proven to improve the well-being and lifespan of an individual.^15^

Aging-associated inflammation and mitochondrial dysfunction are two related phenomena during the aging process,^16–20^, which could be well-connected by succinate accumulation and the subsequent signaling via SUCNR1. Previously, we discovered the significant extracellular signaling functions of succinate which led to increased inflammatory cytokines and the promotion of osteoclastogenesis.^21^ Other studies have also shown that succinate can stimulate the production of pro-inflammatory cytokines, which can contribute to inflammation and disease progression.^22,23^

Importantly, succinate is utilized and produced by bacteria. Interestingly, the relative abundance of the phyla Proteobacteria and Bacteroidetes increases in older subjects^24^ and several genera of these phyla, especially Lipopolysaccharides (LPS)-producing bacteria, are significant contributors to succinate levels in the gut.^25,26^ It is plausible that the increase of succinate and dysbiosis reinforces each other’s impact on the aging process. It is intriguing to explore the role of succinate in the context of dysbiosis and inflammation during aging.

The gut microbiome has been accepted as a contributory factor in aging-related health loss, and extensive researches have explored the connection between aging and gut dysbiosis. The human oral cavity is a complex ecological system harboring a diverse community of microorganisms.^27^ It serves as the gateway to the gut, and growing evidence highlights the importance of oral health for overall well-being. Opportunistic pathogens in the oral cavity can induce systemic inflammation by altering the gut microbiome through the digestion process and there’s evidence that the translocation of oral bacteria to the gut is more likely to occur in aged people.^28–30^ However, the changes and effects of oral microbiota in aging remain poorly understood.

Our previous investigation suggested succinate activation of SUCNR1 exerts a pivotal influence on the immune response to oral pathogens.^31^ It is plausible that aging-related dysbiosis contributes to immune-metabolic processes through succinate and its activation of SUCNR1. In the current study, we investigated the changes in succinate levels, inflammation markers, and oral dysbiosis to evaluate the patterns of aging-related health degradation indicated by autonomously developed periodontal bone loss along aging in the presence and absence of SUCNR1. Importantly, the interaction between inflammation and oral microbiota is influenced by succinate levels. Our metagenomic analysis uncovered a succinate-SUCNR1-driven adaptation in the composition and function of the oral microbiota during aging.

## Results

### Aging-related alveolar bone loss is accompanied by changes in the oral microbiome and an increase in succinate levels

**Fig. 1:**
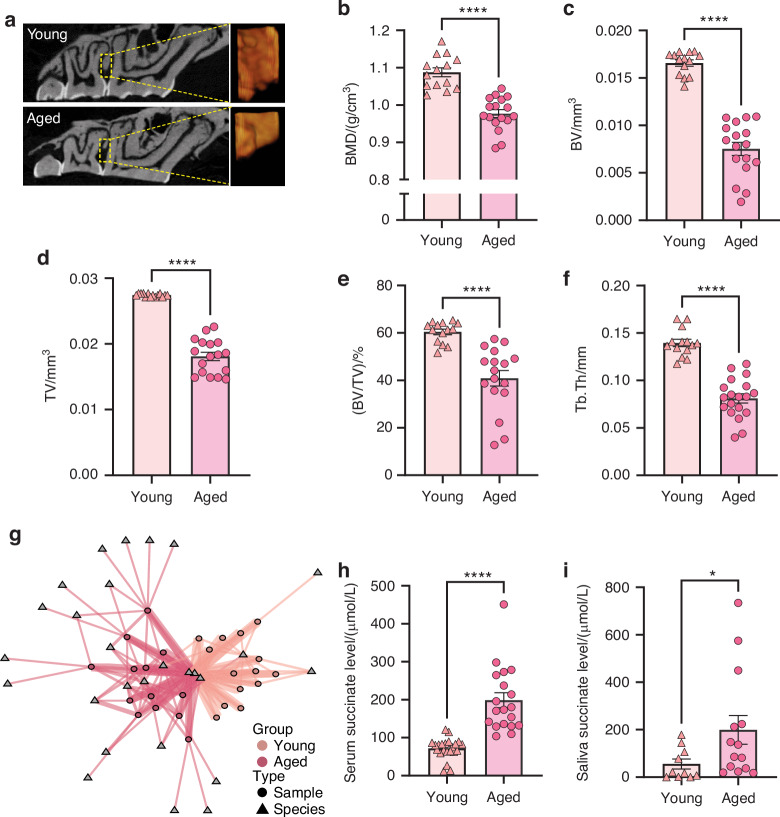
Aging-associated alveolar bone loss is accompanied by shifts in the oral microbiome and changes in succinate levels. **a** Representative μCT images and (**b**–**f**) BMD, BV, TV, BV per TV, and Tb.Th. The analysis with alveolar bone crest regions. Error bars show mean ± SEM. *n* = 14–17, ****P < 0.000 1. **g** Sample-Species interaction network showed a clear partition between young and aged mice. **h** Serum succinate level. Error bars show mean ± SEM. *n* = 19–22, ****P < 0.000 1. **i** Saliva succinate level. Error bars show mean ± SEM. *n* = 10–14, *P < 0.05

We observed a significant alveolar bone recession in wild-type aged mice (2-year-old) by comparing them to their younger counterparts (3-month-old) (Fig. 1a). We compared the alveolar bone volume within consistent depth from the cementoenamel junction (CEJ) across groups. Our finding reveals a notable decrease in bone mineral density (BMD) (Fig. 1b) in the aged mice group, accompanied by alveolar bone loss indicated by the absolute Bone Volume (BV) as well as the ratio of relative BV, i.e., the ratio of BV to Tissue Volume (TV), and the trabecular bone thickness (Tb. Th) (Fig. 1c–f). The sample-species network illustrates different oral microbial compositions between the young and aged mice (Fig. 1g). A noticeable partition was observed between the clusters of young and aged samples, which were distinct from each other. The young mice shared more similarities and formed a tight cluster group with few interconnected species. In contrast, the aged mice have more variation across samples with numerous species being unique to one or two samples (Fig. 1g). Moreover, we observed a statistically significant succinate elevation in the serum of aged mice (Fig. 1h). Intriguingly, this change was mirrored in the oral environment, as evidenced by elevated succinate levels in saliva (Fig. 1i). The concurrent manifestation of compromised alveolar bone phenotype and increased succinate levels in aged animals suggests a potential involvement of succinate and its receptor in oral health during aging. Therefore, we compared aged WT and SUCNR1 knockout mice next.

### Aged-related changes in the oral microbial community composition in the presence and absence of SUCNR1

**Fig. 2:**
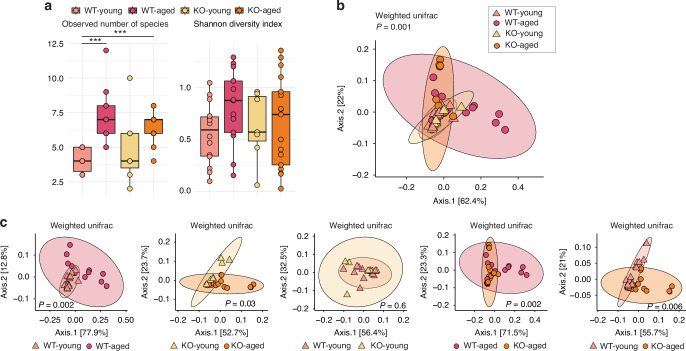
The absence of the SUCNR1 receptor altered the age-related structure of the oral microbial community. **a** Boxplots of alpha diversity measured by the observed number of species and the Shannon diversity index. Significance is shown with asterisks: ***P < 0.005, by Dunn’s pairwise tests. Multiple samples may be represented by a single dot, with possible overlap indicating similar values among samples. **b** Beta diversity measured by Weighted UniFrac distance across four study groups is visualized by PCoA plot. Multiple samples may be represented by a single dot, with possible overlap indicating similar values among samples. **c** Pairwise Beta diversity comparisons measured by Weighted UniFrac distance visualized by PCoA plots. Multiple samples may be represented by a single dot, with possible overlap indicating similar values among samples

We compared oral microbial diversity and composition across different age groups and genotypes of mice to investigate the impact of succinate receptor SUCNR1 on the oral microbiome. Alpha diversity analysis showed that aged mice exhibited higher species richness when compared to young mice, regardless of genotype (Fig. 2a). In WT mice, the observed number of species was significantly higher in the aged group compared to the young group. Although a similar trend was exhibited in the KO mice, the difference was not statistically significant (Fig. 2a). The KO-Aged mice also had a significantly increased number of species compared to the WT-Young mice (Fig. 2a).

Beta diversity analysis was performed using a Weighted UniFrac distance matrix and visualized by PCoA plots to access the overall microbial composition across the four study groups. The all-group comparison indicated significant dissimilarities in the oral bacterial species composition between the study groups (Fig. 2b). Pairwise comparisons showed that WT-Aged and KO-Aged mice significantly differed from their young counterparts (Fig. 2c). No differences between the WT and KO mice were observed when they were young, but the WT-Aged and KO-Aged mice were significantly different from each other, suggesting the WT and KO mice experienced different aging-related changes (Fig. 2c).

**Fig. 3:**
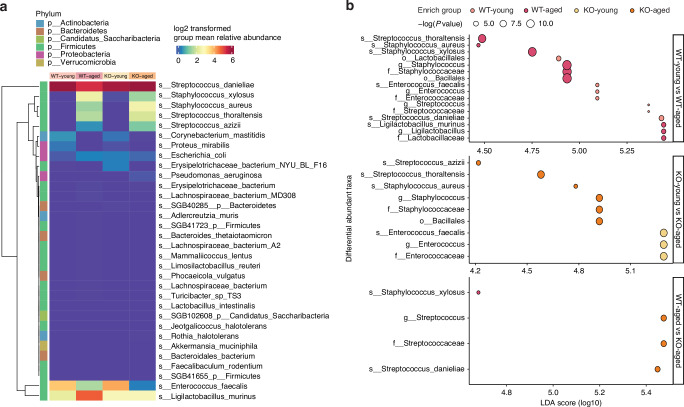
The composition of the oral microbial community altered in aged mice. **a** Hierarchical clustered species level heatmap plotted based on log2 transformed group mean relative abundance. The columns represent the study groups, and the rows represent each species annotated with the corresponding phylum level rank on the side. **b** Dotplots of differentially abundant taxa by pairwise LEfSe analysis. Dot size represents the negative values of the log10 of *P* values, and bigger dots represent smaller *p* values. No differential markers were identified between the WT-Young and KO-Young groups; therefore, the figure does not show the comparison

Hierarchical clustered species level heatmap was plotted based on log2 transformed group mean relative abundance and suggested that regardless of age and genotype, species of the *Firmicutes*, particularly *Staphylococcus danieliae*, were overwhelmingly dominant in all the study groups compared to others (Fig. 3a). Notably, WT-Young and KO-Young mice had highly similar species relative abundance profiles, which differed from those of their older counterparts. However, while WT-Aged and KO-Aged mice exhibited similar patterns, there were still notable differences between them (Fig. 3a). Lefse analysis identified statistically enriched or depleted taxa in a pairwise fashion (Fig. 3b). WT mice exhibited the highest number of differentially abundant taxa when comparing WT-Young and WT-Aged groups (Fig. 3b). In KO mice, only four species were found to be differentially abundant, among which *Streptococcus azizii, Streptococcus thoraltensis*, and *Staphylococcus aureus* were enriched in the KO-Aged group. When comparing the aged groups across mouse genotypes, *Staphylococcus xylosus* was enriched in the WT-Aged group, and *Staphylococcus danieliae* was enriched in the KO-Aged group. There were no statistically significant differential taxonomic markers found when comparing the two young mice groups.

### Analysis at the gene-family level revealed that the absence of SUCNR1 leads to reduced age-related changes in functional potential

**Fig. 4:**
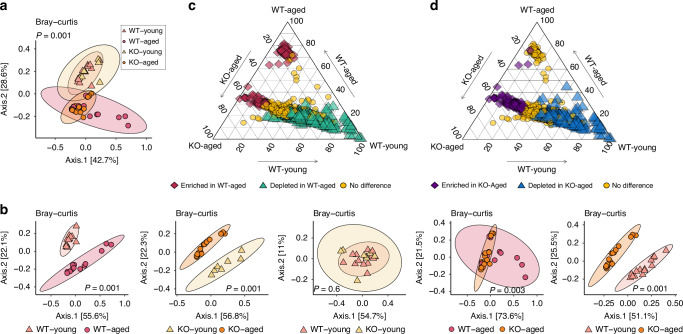
Reduced age-related changes in functional potential at the gene family level occur in the absence of SUCNR1. **a**) Microbial gene family Beta diversity comparisons measured by Bray-Curtis dissimilarity across four study groups on PCoA plot. Multiple samples may be represented by a single dot, with possible overlap indicating similar values among samples. **b** Pairwise microbial gene family Beta diversity comparisons measured by Bray-Curtis dissimilarity on PCoA plot. Multiple samples may be represented by a single dot, with possible overlap indicating similar values among samples. **c** Ternary diagram displays the gene family distribution across the WT-Young, WT-Aged, and KO-Aged groups. Each point represents a gene family. The color and shape of the point represent the MaAsLin2 test results comparing the WT-Aged group to the WT-Young group. Each apex represents a study group, and points that are closer to a group have a higher group mean relative abundance of that gene than the other groups. **d** The Ternary diagram displays the gene family distribution across the WT-Young, WT-Aged, and KO-Aged groups. Each point represents a gene family. The color and shape of the point represent the MaAsLin2 test results comparing the KO-Aged group to the WT Young group. Each apex represents a study group, and points that are closer to a group have a higher group mean relative abundance of that gene than the other groups

To explore the functional implications of our findings, the Uniref90 gene family relative abundance across groups was assessed by Bray-Curtis beta diversity analysis. We found that the young groups formed clearly separate clusters from the aged groups, indicating that gene family diversity was altered in both WT and KO aged mice (Fig. 4a). Pairwise analysis confirmed that both the WT-Aged and KO-Aged mice were different from their young counterparts, but, significantly, they also differed from each other (Fig. 4b). As no significant differences were observed between the WT-Young and KO-Young mice, ternary diagrams were generated to display the gene family distribution across the WT-Young, WT-Aged and KO-Aged groups (Fig. 4c, d). Differentially enriched or depleted gene families in the aged groups compared to the WT-Young group were then identified by MaAsLin2 tests and colored on the ternary diagrams. There was a notable number of gene families significantly enriched in the WT-Young group but depleted in both aged groups, and an enrichment set of gene families in both WT-Aged and KO-Aged mice, indicating certain shared functional features between them. While the WT-Aged mice showed enrichment or depletion in certain gene families that overlapped with those in the KO mice, they also exhibited a unique set of enriched gene families not found in the KO mice. This suggests a distinct functional pattern in the WT-Aged mice compared to the KO-Aged mice (Fig. 4c, d). To further investigate the functional categories of the genes, we regrouped the Uniref90 gene families into MetaCyc reactions and again performed beta diversity analysis across groups (Fig. S1a). After regrouping, the significant differences previously observed persisted in the WT mice. However, the KO-Young and KO-Aged groups no longer showed statistical differences, indicating a shared functional context for the differentially abundant genes in KO mice, regardless of age (Fig. S1b).

### Functional potential is preserved at the pathway level in the absence of SUCNR1

**Fig. 5:**
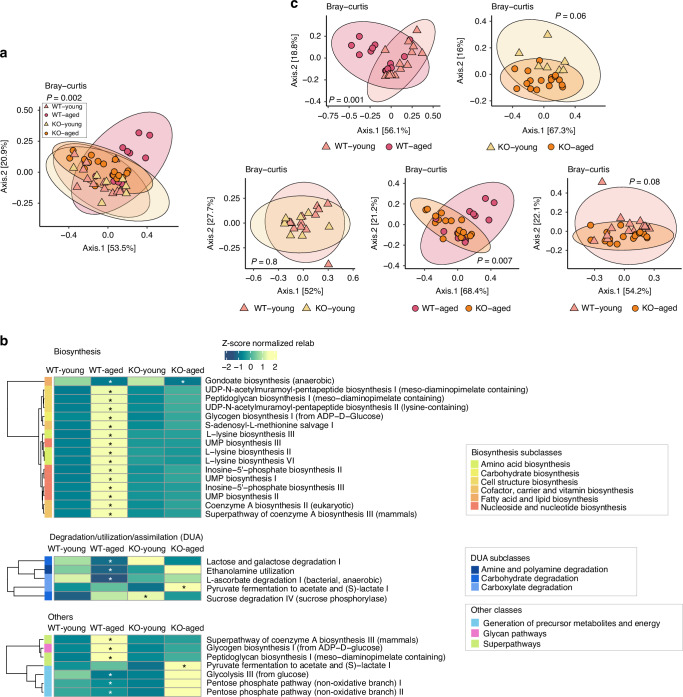
Age-related functional potential is preserved at the pathway level in the absence of SUCNR1. **a** Metacyc pathway Beta diversity comparisons measured by Bray-Curtis dissimilarity across four study groups on PCoA plot. Multiple samples may be represented by a single dot, with possible overlap indicating similar values among samples. **b** Pairwise Metacyc pathway Beta diversity comparisons measured by Bray-Curtis dissimilarity on PCoA plot. Multiple samples may be represented by a single dot, with possible overlap indicating similar values among samples. **c** Hierarchical clustered heatmap based on z-score normalized group mean relative abundance of the significant Metacyc pathways identified by MaAsLin2 tests (study group as fixed effect, WT young as reference level). The MaAsLin2 corrected significance is shown with asterisks: qval < 0.1

Although substantial differences with aging were observed at the gene family level in the KO mice, these differences did not translate into significant variations in the reactions forming pathways at broader functional levels. Further analysis confirmed this, with PCoAs of Bray-Curtis beta diversity at the pathway level for WT-Young, KO-Young, and KO-Aged showing similar significance patterns to those observed at the reaction level (Fig. 5a, b). The WT and KO young groups remained similar in terms of all the functional features investigated. The WT-Aged mice significantly differed from the WT-Young mice and the KO-Aged mice, while the KO-Aged mice were similar to both KO-Young and WT-Young groups (Fig. 5b). Significantly differentially enriched or depleted Metacyc pathways identified by MaAsLin2 tests comparing the WT-Aged, KO-Young, and KO-Aged groups to the WT-Young group were plotted on a hierarchical clustered heatmap based on z-score normalized relative abundance (Fig. 5c). The WT-Aged mice generally displayed a significantly different profile compared to the other three groups. Specifically, 15 biosynthesis pathways were uniquely enriched in the WT-Aged group, which were involved in the biosynthesis of amino acids, carbohydrates, cell structure, cofactors, carriers, vitamins, and nucleosides and nucleotides. The gondoate biosynthesis (anaerobic) pathway, involved in fatty acid and lipid biosynthesis, was depleted in the aged mice of both WT and KO genotypes. Three degradation pathways were found to be solely depleted in the WT-Aged mice. The pyruvate fermentation to acetate and (S)-lactate I pathway was enriched in KO-Aged mice, and the sucrose degradation IV (sucrose phosphorylase) pathway was enriched in the KO-Young mice.

### Reduced age-related bone loss and inflammation in the absence of SUCNR1

**Fig. 6:**
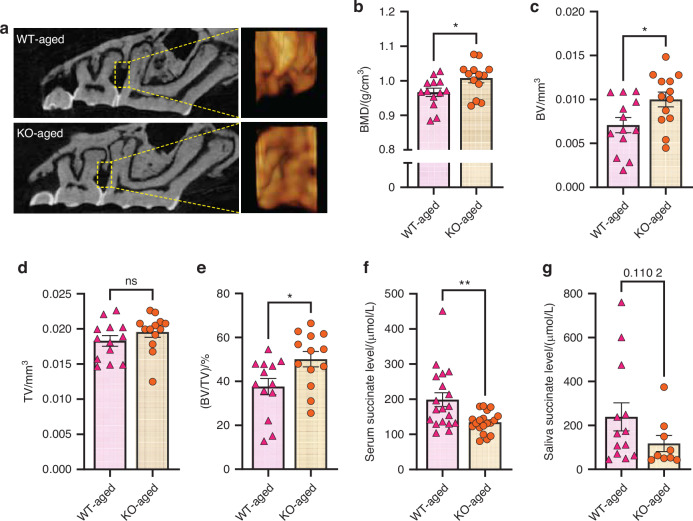
Reduced age-related bone loss and inflammation in the absence of SUCNR1. **a**–**e** Representative μCT images and analysis with alveolar bone crest regions in aged mice. Error bars show mean ± SEM. *n* = 13, *P < 0.05. **f**–**g** Succinate levels in serum and saliva of aged mice. Error bars show mean ± SEM. *n* = 9–19, **P < 0.005

We observed significantly higher BMD, BV, and BV/TV ratios in aged KO mice compared to aged WT mice, with a trend towards increased tissue volume in the aged KO mice. This suggests that SUCNR1 ablation effectively mitigates age-related bone loss (Fig. 6a–e). Additionally, we noted a relatively lower accumulation of succinate in the serum (Fig. 6f) and saliva (Fig. 6g) of aged KO mice, suggesting that the absence of SUCNR1 reduced aging-related elevation of succinate.

**Fig. 7:**
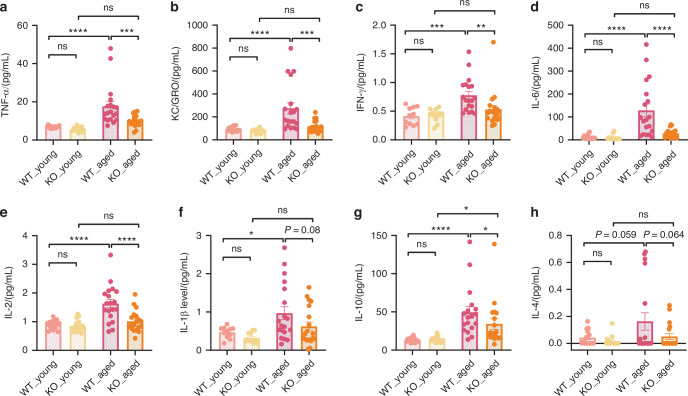
Cytokine levels in young and aged WT and KO mice. **a** TNF-α, **b** KC/GRO, **c** IFN-γ, **d** IL-6, **e** IL-2, **f** IL-1β, **g** IL-10, and **h** IL-4 level were compared between the indicated groups. Error bars show mean ± SEM. *n* = 14–19, *P < 0.05, **P < 0.005, ***P < 0.001, and ****P < 0.000 1

Serum samples from both young and aged mice were analyzed using an MSD pro-inflammatory assay kit. We found that KO-Aged mice had significantly lower levels of pro-inflammatory cytokines, including Tumor Necrosis Factor-alpha (TNF-α), Keratinocyte-derived Chemokine/ Growth-regulated Oncogene (KC/GRO), Interferon- gamma (IFN-γ), Interleukin- 6 (IL-6), and Interleukin-2 (IL-2), compared to WT-aged mice (Fig. 7a–e). Additionally, Interleukin- 1 beta (IL-1β) levels showed a trend toward reduction in KO-Aged mice compared to WT-Aged mice (Fig. 7f), though this difference was not statistically significant. Notably, two key anti-inflammatory cytokines, Interleukin-10 (IL-10) and Interleukin- 4 (IL-4), were also upregulated in WT-Aged mice (Fig. 7g, h). This increase may represent a compensatory mechanism to maintain immune homeostasis and regulate inflammation. These findings suggest that SUCNR1 plays a crucial role in regulating the inflammatory environment associated with aging.

### Distinct correlation networks between succinate levels and biological pathways are observed with and without SUCNR1

**Fig. 8:**
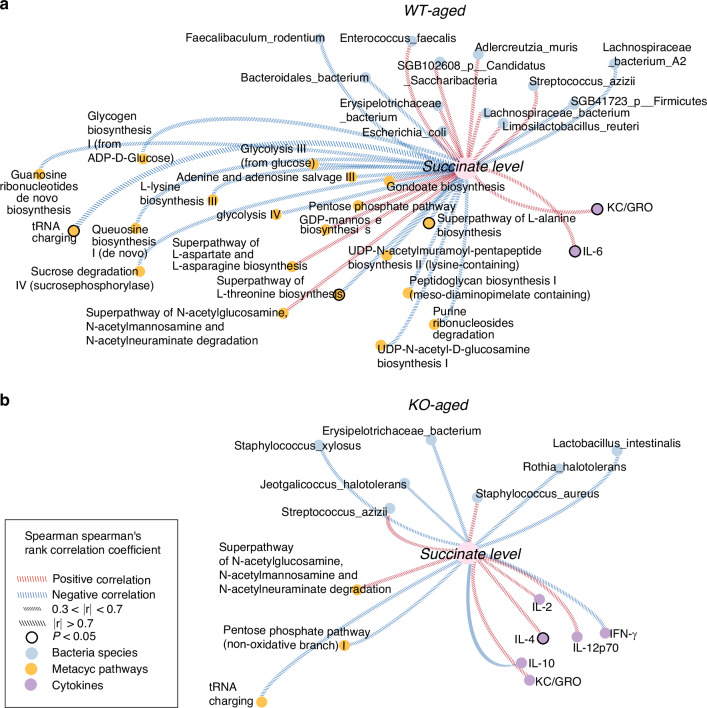
Distinct correlation networks between succinate levels and biological pathways. **a** WT-Aged and **b** KO-Aged mice showed distinct correlation networks between succinate levels, microbial species, pathways, and levels of cytokines

Correlation networks were generated to explore any significant associations between succinate levels and bacterial species relative abundance, pathway relative abundance, and pro-inflammatory cytokine levels in WT-Aged and KO-Aged mice. There were 20 pathways that demonstrated pronounced associations (|r|>0.3) with succinate levels in WT-Aged mice (Fig. 8a). In contrast, KO-Aged mice exhibited only three moderate pathway associations, suggesting the absence of the host succinate receptor might have disrupted the metabolic effects of elevated succinate on the oral microbiome (Fig. 8b). Notably, in WT-Aged mice, the superpathway of L-alanine biosynthesis, superpathway of L-threonine biosynthesis, and tRNA charging pathways had strong and statistically significant negative correlations with succinate levels (|r|= 0.61, 0.63, 0.78, *P* = 0.04, 0.03, 0.005, respectively). The superpathway of N-acetylglucosamine, N-acetylmannosamine, and N-acetylneuraminate degradation, gondoate biosynthesis (anaerobic) and GDP-mannose biosynthesis pathways were positively correlated with succinate levels in the WT-Aged mice, suggesting a noticeable tendency to increase in pathway relative abundance with succinate elevation (0.4 < |r|< 0.6). The tRNA charging pathway also showed a moderate negative correlation with succinate level in KO-Aged mice, and the superpathway of N-acetylglucosamine, N-acetylmannosamine, and N-acetylneuraminate degradation also showed a moderate positive correlation with succinate level in KO-Aged mice, but none of the correlations between pathway and succinate levels were statistically significant. The elevated succinate levels in WT-Aged mice were significantly correlated with increased KC/GRO and IL-6 levels (|r| = 0.59, 0.60, *P* = 0.05, 0.04, respectively) (Fig. 8a). However, in KO-Aged mice, succinate level was only significantly correlated with IL-4 (|r| = 0.6, *P* = 0.01).

## Discussion

We demonstrated that aging-associated alveolar bone loss is linked with oral dysbiosis and increased succinate levels in both serum and saliva. Additionally, in the absence of succinate/SUCNR1 signaling, the oral microbiome of aged mice more closely resembles that of young mice, with distinct networks emerging between microbiome features and succinate levels. This is accompanied by a reduction in age-associated succinate elevation, bone loss and inflammatory response during aging in the absence of SUCNR1.

Succinate binds to its G-protein-coupled receptor SUCNR1, which enhances osteoclast activity. This interaction stimulates the release of pro-inflammatory cytokines, promoting inflammation, leading to tissue damage, and resulting in increased bone resorption.^21^ Our previous research has shown that succinate and SUCNR1 signaling are crucial in the progression of periodontitis, establishing a connection between succinate levels, host signaling, and dysbiosis.^31^ The mitigated aging-related bone loss and inflammation in aged SUCNR1 KO mice could also be contributed by a significant reduction of succinate elevation.

The oral microbiome gradually changes and shifts during the development of the body in response to aging,^32^ diet,^33^ and other lifestyle-related factors (e.g., oral hygiene practices, smoking, and alcohol consumption.^34–36^ Maintaining a balanced oral microenvironment is not only crucial to oral health but may also have broader implications on the overall health of the host. Oral dysbiosis, although not necessarily to be an indicator of aging, increases in prevalence with age,^37,38^ and is highly associated with many health problems in older populations, including diabetes,^39^ periodontitis Alzheimer’s,^40^ and cardiovascular diseases.^41^

The mouse oral microbiome was dominated by *S. danieliae* and a few other species.^42–45^ Congruent with these studies, in our current study, *S. danieliae* was significantly enriched in both the KO-Aged and WT-Young group compared to the WT-Aged group, suggesting the absence of host succinate singling, due to low succinate levels and/or SUCNR1 KO, maintained a more youthful oral microbiome composition. Interestingly, *S. danieliae* is a microaerophile, and its depletion may indicate an expansion of anaerobic niches. *S. xylosus* was significantly enriched in the WT-Aged group compared to KO aged mice without host succinate signaling. *S. xylosus* has been reported to be enriched and more transcriptionally active in induced periodontal disease in mice,^43^ indicating that age-associated host succinate signaling may enrich periodontal pathogens.

Aging presents a predisposition to various oral complications, including bone loss, inflammation, and dysbiosis. As one ages, the body experiences a continuous decline in metabolic capabilities and immune responses. Such a decline is a systematic downturn affecting the metabolic functions of the residual microorganisms, including oral microbes, to feed in nutrients and produce essential metabolites for cell functioning,^32^ making the host more susceptible to inflammation and dysbiosis.

The WT-Aged group showed multiple negative, pronounced associations between MetaCyc pathways and succinate levels. These pathways tended to be involved in biosynthesis pathways intimately connected with the central carbon metabolism and the TCA cycle metabolite succinate (i.e., glycolysis/gluconeogenesis, TCA cycle). In the case of excess succinate in the WT-Aged animal, microbiota may utilize succinate for anaplerotic reactions connected to the TCA cycle and provide carbon atoms for gluconeogenesis from TCA cycle-derived oxaloacetate. For example, glycolysis III and IV and sucrose degradation pathways all had negative associations with succinate levels. In addition, the Pentose phosphate pathway (PPP), which can utilize gluconeogenic metabolites (e.g., glucose-6-phosphate), is responsible for the production of reducing equivalents for biosynthesis and nucleotide precursors (e.g., ribulose 5-phosphate) and showed positive associations with succinate levels concomitant with a negative association with purine ribonucleosides degradation. Conversely, in KO-Aged animals a negative association is found with the non-oxidative branch of the PPP, responsible for nucleotide precursor’s generation (e.g., ribulose-5-phosphate), concomitant with positive associations with the fermentation of the glycolytic product pyruvate. Potentially in support of a WT-Aged community primed to utilize succinate to fuel TCA cycle and gluconeogenesis activity, degradation pathways for lactose (results in glucose production), ethanolamine utilization (results in acetyl-CoA production),^46^ and L-ascorbate degradation (results in ribulose 5-phosphate production through the PPP)^47^ were depleted in the oral microbiome of WT-Aged animals.

In conclusion, our findings highlight the critical interplay between succinate signaling, the oral microbiome, and the aging process. The observed reduction in age-related bone loss and inflammation, along with a younger-like oral microbiome function in the absence of succinate signaling, underscores the potential of targeting the succinate-SUCNR1-oral microbiome axis to alleviate the effects of inflammaging on both oral and systemic health.

## Materials and Methods

### Animals

All animal experiments in this study received approval from the Institutional Animal Care and Use Committee (IACUC) and adhered to the guidelines from the Division of Laboratory Animal Resources. The animals were housed in Specific pathogen-free units at the New York University animal facility. All mice used in this study were of C57BL/6 J (B6) background. The SUCNR1 flox mice were developed using a TurboKnockout gene targeting strategy at Cyagen and Taconic Companies, and the global knockout of SUCNR1 (KO) mice was subsequently generated through breeding with the EIIa-Cre mice (Jackson Laboratory, Bar Harbor, ME, USA) as previously described.^48^ Aging models: Wild-type (WT) and SUCNR1 knockout (KO) mice were genotyped and maintained for 3 months (young) and 24 months (aged) in a pathogen-free facility with ad libitum access to food and water without additional interventions. Sterile swabs were employed for the collection of oral microbiota from the mice before euthanasia, and the swab tips were snap-frozen in TE buffer for subsequent procedures (Puritan, ME, USA. Catalog number: 25-800-A50). Mice serum samples were collected at the endpoint. All the procedures were approved by the New York University IACUC committee.

### Three-dimensional micro-computed tomography analysis

Maxillae were fixed in 10% buffered formalin for 48 h, triple-washed with PBS, and stored in 70% ethanol for later scan. The fixed samples were scanned using a SkyScan 1172 high-resolution scanner (Bruker microCT, Kontich, Antwerp, Belgium) at 60 kV, 167 μA, and a spatial resolution of 9.7 μm. Reconstruction was performed with NRecon (Bruker), followed by reorientation using the DataViewer (Bruker) for further analysis. We selected the alveolar bone up to 0.58 mm (60 slides) from the midpoint of cementoenamel junctions (CEJ) of first and second molars as volume of interest (VOI) within 0.27 mm diameter round shape regions. Bone mineral density (BMD) and morphometric parameters such as bone volume (BV), tissue volume (TV), and relative bone volume (BV/TV) were analyzed in CTAn (Bruker). The 3D image of the alveolar bone was generated using CTVox. Comparison between two groups was analyzed using Student’s t-test.

### Succinate colorimetric assay

We conducted this assay using a colorimetric assay kit from Abcam (Kit ab204718, Abcam, Cambridge, United Kingdom) to measure the succinate level in mice serum and saliva. For serum collection, mouse blood was collected via cardiac puncture and transferred to BD microtainer serum separator tubes. The tube was left at room temperature for a minimum of 30 minutes, followed by 10-minute centrifugation at 200 g to facilitate the separation of the serum. Serum samples were aliquoted immediately upon separation and snap-frozen till use. For saliva collection, mice were stimulated with 0.45 mg/kg pilocarpine via intraperitoneal injection. We placed sterile Puritan Calgiswab tip in the mouse’s mouth to absorb the saliva, then transferred the swab into a 0.5 mL tube with a hole in the bottom. The tube was then inserted into a 1.7 mL tube, and centrifuged at 200 g for 5 minutes to allow the saliva been transferred into the 1.7 mL tube. Saliva samples were aliquoted immediately upon separation and snap-frozen till use. The assay was performed in accordance with the manufacturer’s protocol.

### Cytokine measurement in mouse serum

We used Meso Scale Discovery V-PLEX Pro-inflammatory Panel 1 Mouse Kit to determine pro-inflammatory biomarkers including IL-1β, TNF-α, KC/GRO, IFN-γ, IL-6, and IL-2. Briefly, whole blood was collected and allowed to clot by leaving it undisturbed at room temperature for 30 minutes. The clot was removed by centrifuging at 1 000–2 000 × *g* for 10 minutes in a refrigerated centrifuge. The resulting supernatant is designated serum and was frozen immediately until the assay. The samples were thawed and diluted 2 times using the sample diluent buffer provided in the kit. The assay was conducted according to the manufacturer’s instructions as described in our previously published study.^31^ Data were analyzed using ANOVA with Tukey’s post hoc test.

### DNA library construction and shotgun metagenomic sequencing

Mouse oral swabs were subjected to DNA extraction using a Qiagen QIAamp PowerFecal Pro DNA kit according to the manufacturer’s instructions. Extracted DNA was quantified using Quant-iT PicoGreen dsDNA Assay Kit (Invitrogen, Waltham, MA, USA) and checked for purity by NanoDrop Spectrophotometer. DNA samples were normalized to 0.8 ng/μL prior to performing library construction. Shotgun metagenomic DNA libraries were constructed using Illumina DNA Prep containing the Illumina Purification Beads and IDT for Illumina DNA/RNA UD Indexes according to the manufacturer’s instructions for samples <100 ng DNA, using 12 PCR cycles (Illumina, San Diego, CA, USA). Libraries were pooled in equimolar amounts and sequenced on an Illumina NextSeq 1000 platform with a 300-cycle (2 × 150) P2 reagent kit (V3). Negative controls were handled exactly as samples and consisted of water and buffer used in DNA extractions and dilutions. ZymoBIOMICS Fecal Reference with TruMatrix Technology (ZYMO Research, Irvine, CA, USA) was included as a sequencing quality control.

### Bioinformatics analysis of sequencing data

Paired-end raw sequencing reads in FASTQ format were quality controlled using KneadData pipeline (https://huttenhower.sph.harvard.edu/kneaddata/), including the trimming of adapter sequences, overrepresented sequences and low-quality reads using Trimmomatic,^49^ then host DNA contaminating reads were checked and removed using bowtie2^50,51^ against the mouse (C57BL) reference database. In total, 176 FASTQ files from 88 mice oral swab samples were processed. Samples with less than 250k pass-filtering reads were excluded from further analysis. Retained samples (WT-Young *n* = 14, WT-Aged *n* = 13, KO-Young *n* = 7, and KO-Aged *n* = 17) have a mean sequence depth of 1 729 439 reads. Processed metagenomic reads were then classified using MetaPhlAn 4.0.4 for microbial community taxonomic profiling.^52^ HUMAnN 3.6 was used to profile the abundance of UniRef90 gene families and microbial metabolic pathways in the samples.^53^ The profiled functional features in reads per kilobase (RPK) units were transformed to relative abundance. Further, gene families were regrouped into the MetaCyc Reactions using the utility scripts within HUMAnN 3.7.^53^

### Downstream statistical analysis

Taxonomic and functional profiles were imported into phyloseq objects^54^ to perform downstream analysis in R v4.2.2. Alpha diversity metrics were calculated using the microbiome package v1.20.0. A Kruskal-Wallis test followed by Dunn’s pairwise test was used to identify the significant differences in Alpha Diversity indices across and between groups. Beta diversity analysis was performed on Weighted Unifrac distance metrics and visualized by principal coordinate analysis (PCoA), and the statistical difference was accessed by Permutational multivariate analysis of variance (PERMANOVA) using the Vegan r package v2.6-4. The log2-transformed species-level group mean relative abundance was plotted and grouped by hierarchical clustering using the pheatmap r package v1.0.12. Differentially abundant taxa between groups were identified by pair-wised Linear discriminant analysis effect size (LEfSe)^55^ analysis, and significantly enriched species were determined with LDA scores >3. Differentially abundant gene families, MetaCyc reactions and pathways were identified by Multivariate Associations by Linear models (MaAsLin2).^56^ An adjusted *p*-value of <0.1 was considered significant for all MaAsLin2 tests performed in functional analysis. All the functional features included in the analysis must be present in more than 75% of samples in any comparative groups. In total, 140 835 microbial UniRef90 gene families were identified, and 2 736 of them were present in more than 75% of samples, and unmapped and ungrouped functional features were not included in the analysis. Beta diversity analysis for all the functional features was performed on Bray-Curtis dissimilarity metrics and visualized by PCoA and the statistical difference was accessed as previously stated. Group distribution of the 2 736 gene families was accessed on a on ternary diagram prepared using prep_ternary function within the microbiomeutilities r package and visualized using the ggtern r package v3.4.2. Differential pathway mean relative abundance were z-score transformed and plotted on a heatmap grouped by each Metacyc class hierarchy. The Spearman correlation coefficient was calculated to assess the relationships among the relative abundance of the bacterial species, Metacyc pathways, inflammatory cytokine levels, and succinate levels using the rstatix package v0.7.2. Correlation networks were generated using the Cytoscape v3.10.1.

## Supplementary information

Supplementary Information

Supplementary Information

## Data Availability

The raw sequencing data from this study is publicly accessible under the NCBI BioProject accession number PRJNA1196948. Data used for subsequent analyses, such as raw feature profiles, quality control metrics, scripts for generating figures, and other supplementary materials, can be found in our GitHub repository at https://github.com/Fangxi-Xu/Succinate_Aging_Metag.
