# Supplementary material for: Succinate modulates oral dysbiosis and inflammation through a succinate receptor 1 dependent mechanism in aged mice
Source: Int J Oral Sci. 2025 Jun 10;17:47. doi: 10.1038/s41368-025-00376-6 (PMC12152193; doi:10.1038/s41368-025-00376-6)
Supplement: Supplementary file 1 — Supplementary Information [file 41368_2025_376_MOESM1_ESM.docx]

**Fig. S1: Genefamilies regrouped into Metacyc reactions.**

(a) Microbial Metacyc reaction Beta diversity comparisons measured by Bray-Curtis dissimilarity across four study groups on PCoA plot. Multiple samples may be represented by a single dot, with possible overlap indicating similar values among samples.

(b) Pairwise microbial Metacyc reaction Beta diversity comparisons measured by Bray-Curtis dissimilarity on PCoA plot. Multiple samples may be represented by a single dot, with possible overlap indicating similar values among samples.
