# Supplementary material for: Succinate modulates oral dysbiosis and inflammation through a succinate receptor 1 dependent mechanism in aged mice
Source: Int J Oral Sci. 2025 Jun 10;17:47. doi: 10.1038/s41368-025-00376-6 (PMC12152193; doi:10.1038/s41368-025-00376-6)
Supplement: Supplementary file 2 — Supplementary Information [file 41368_2025_376_MOESM2_ESM.pdf]

Fig. S1

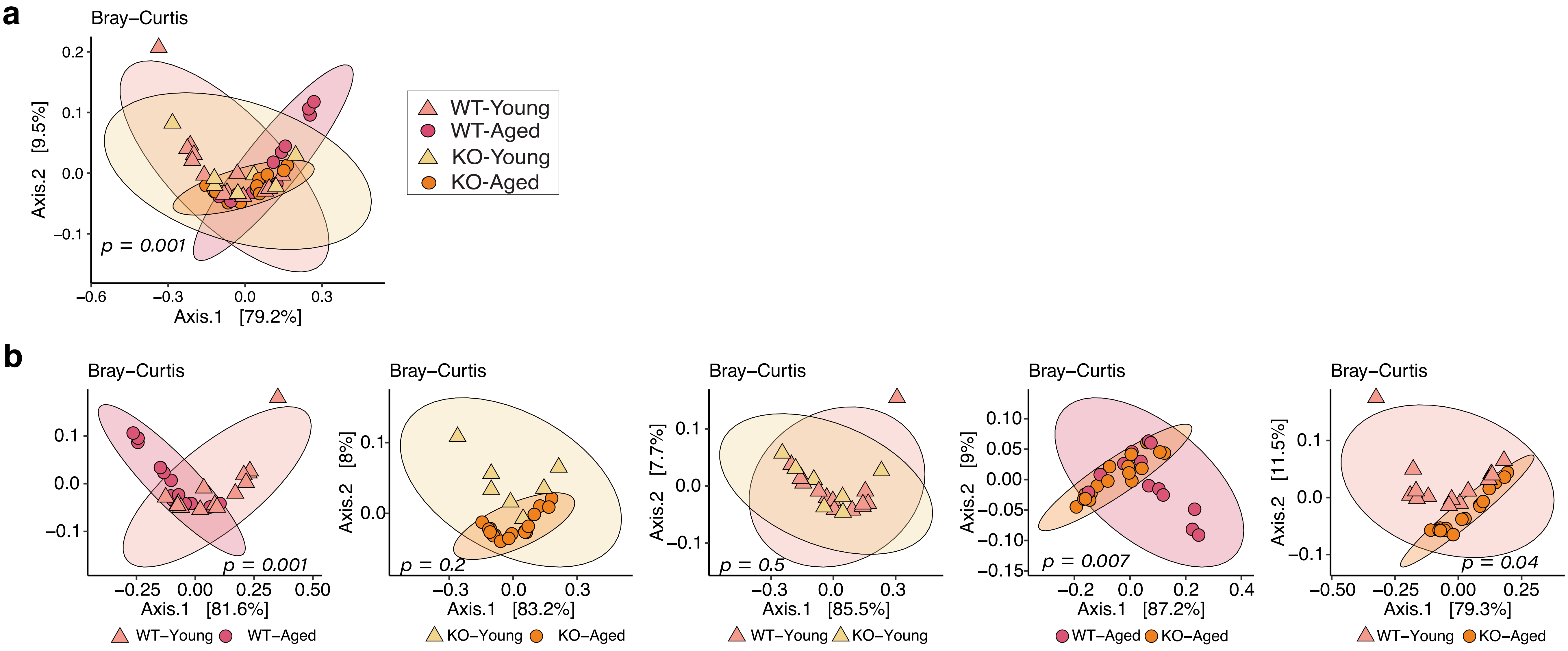
a
Bray−Curtis
WT-Young
WT-Aged
KO-Young
KO-Aged
p = 0.001
Axis.1 [79.2%]
Axis.2 [9.5%]
0.2
0.1
0.0
−0.1
−0.6
−0.3
0.0
0.3
b
Bray−Curtis
p = 0.001
Axis.1 [81.6%]
−0.25
0.00
0.25
0.50
WT−Young
WT−Aged
Bray−Curtis
p = 0.2
Axis.1 [83.2%]
Axis.2 [8%]
KO−Young
KO−Aged
Bray−Curtis
p = 0.5
Axis.1 [85.5%]
Axis.2 [7.7%]
WT−Young
KO−Young
Bray−Curtis
p = 0.007
Axis.1 [87.2%]
Axis.2 [9%]
0.10
0.05
0.00
−0.05
−0.10
−0.15
−0.2
0.0
0.2
0.4
WT−Aged
KO−Aged
Bray−Curtis
p = 0.04
Axis.1 [79.3%]
Axis.2 [11.5%]
−0.50
−0.25
0.00
0.25
WT−Young
KO−Aged

Fig. S1: Genefamilies regrouped into Metacyc reactions. (a) Microbial Metacyc reaction Beta diversity comparisons measured by Bray-Curtis dissimilarity across four study groups on PCoA plot. Multiple samples may be represented by a single dot, with possible overlap indicating similar values among samples. (b) Pairwise microbial Metacyc reaction Beta diversity comparisons measured by Bray-Curtis dissimilarity on PCoA plot. Multiple samples may be represented by a single dot, with possible overlap indicating similar values among samples.
